# Quality of Life After Laser Vision Correction: A Systematic Review and Meta-Analysis

**DOI:** 10.1155/joph/8833830

**Published:** 2025-01-06

**Authors:** Alireza Peyman, Matin Irajpour, Maryam Yazdi, Farzaneh Dehghanian, Pegah Noorshargh, Yasaman Broumand, Farnaz Fatemi, Mohsen Pourazizi

**Affiliations:** ^1^Department of Ophthalmology, Isfahan Eye Research Center, Isfahan University of Medical Sciences, Isfahan, Iran; ^2^Child Growth and Development Research Center, Research Institute for Primordial Prevention of Non-Communicable Disease, Isfahan University of Medical Sciences, Isfahan, Iran; ^3^Noor Research Center for Ophthalmic Epidemiology, Noor Eye Hospital, Tehran, Iran

**Keywords:** laser in Situ keratomileusis, laser vision correction, photorefractive keratectomy, quality of life, refractive surgical procedures

## Abstract

**Purpose:** To analyze patients' quality of life (QOL) after laser vision correction (LVC) from a worldwide literature review.

**Methods:** Studies of prospective or cross-sectional design which evaluated QOL in patients after LVC and compared that to preoperative values or a matched group of emmetropes were included. The Web of Science, PubMed, Scopus, and ProQuest were searched for relevant articles published until February 2024. The fixed- or random-effects models were used to estimate the weighted mean difference (WMD) for postoperative QOL changes. Meta-regression was conducted for adjusting the effects of potential confounders.

**Results:** A total of 11 peer-reviewed articles (1753 patients) were included in the study. LVC improved QOL of patients at one (SMD = 0.38, 95% CI: 0.15, 0.60), three (SMD = 1.03, 95% CI: 0.55, 1.50), and six months after surgery (SMD = 0.71, 95% CI: 0.30, 1.11). In meta-regression analysis, QOL improvement was lower in older patients compared to younger ones (*β* = −0.06, 95% CI: −0.11, −0.01). Also, no statistically significant difference was noted while comparing QOL in post–laser refractive surgery patients and emmetropes (SMD = −0.44, 95% CI: −0.95, 0.07).

**Conclusion:** Patients undergoing LVC experience significant improvements in QOL, particularly in younger subjects, and achieve comparable QOL to individuals with emmetropia.

## 1. Introduction

Several methods have been developed for treating refractive errors, such as spectacles, contact lenses, and orthokeratology [1]. However, laser vision correction (LVC) is one of the most common ophthalmologic operations performed worldwide [2, 3]. Global demand for refractive surgery has increased in the past decade, and it is expected to rise further [4–8]. A meta-analysis surmised that 95.4% of patients were satisfied with their visual acuity (VA) after laser in situ keratomileusis (LASIK) [9]. The outcome of refractive surgery has usually been characterized by objective standard clinical measurements, such as postoperative uncorrected VA (UCVA), residual refractive error [10], keratometry, contrast sensitivity, corneal high-order aberrations, nerve fiber regeneration, centration of the treatment zone, and corneal biomechanical properties [11–14]. A rather less emphasized subjective measure is patient satisfaction, which has been used to rate hospitals, health plans, and individual physicians. Although the objective measures provide important information, they do not necessarily correlate well with patients' postoperative subjective impressions and visual improvement [15–17]. Quality of life (QOL) questionnaires should be used to measure the results of surgery subjectively, and many of these have been developed [18]. Recently, these subjective patient-based methods such as QOL questionnaires are gaining popularity as methods of assessing the outcome of refractive surgeries [19, 20].

LVC surgeries have been internationally recognized since the late 1980s, with the introduction of photorefractive keratectomy (PRK), which was approved by FDA in 1995. LASIK, introduced in the early 1990s, quickly gained global acclaim for its efficiency and comfort, while SMILE, developed in 2011, is a more recent method [21, 22]. Also more and more patients are opting for refractive surgery as a means of spectacles independence; hence, many QOL questionnaires have been developed for this exact purpose [11]. The following study aims to analyze patients' QOL after LVC using a worldwide literature review.

## 2. Methods

### 2.1. Setting and Search Strategy

The study protocol adhered to the guidelines outlined in the Preferred Reporting Items for Systematic Reviews and Meta-Analysis (PRISMA) 2020 statement. This study has been registered in Isfahan University of Medical Sciences Research Ethics Committee (IR.ARI.MUI.REC.1401.083).

A comprehensive, systematic search was carried out across various databases, including PubMed (National Library of Medicine), Scopus, Web of Science, and Embase, up until February 2024. An expert team conducted preliminary searches to identify pertinent search terms, refine search strategies, and determine relevant information sources. The search strategy incorporated keywords and related MeSH terms:

(“*Quality of Life*” OR “*QOL*” OR “*Health-Related Quality of Life*” OR “*HRQOL*”) AND (“*Refractive Error*” OR “*Myopia*” OR “*Nearsightedness*” OR “*Hyperopia*” OR “*Farsightedness*” OR “*Astigmatism*” OR “*Presbyopia*” OR “*Anisometropia*”) AND (“*Photorefractive Keratectomy*” OR “*PRK*” OR “*Refractive surgery*” OR “*Refractive Surgical Procedures*” OR “*Corneal Surgery*” OR “*Lase- assisted* In Situ *Keratomileusis*” OR “*Keratomileusis, Laser* In Situ” OR “*LASIK*” OR “*Small Incision Lenticule Extraction*” OR “*SMILE*” OR “*Laser-assisted Subepithelial Keratectomy*” OR “*LASEK*”)

Furthermore, we conducted a review of the references cited in primary articles to identify additional studies related to the topic.

### 2.2. Eligibility Criteria

We incorporated original studies that evaluated QOL in patients after LVC and compared that to preoperative values or a matched group of emmetropes. Excluded from consideration were studies involving patients with refractive surgery plans other than corneal procedures (e.g., phakic intraocular lens and refractive lens exchange), conference articles, abstracts or protocols, narrative and systematic reviews, case reports, and consensus opinions. In addition, studies published in languages other than English were also excluded. Moreover, studies that failed to report necessary information for calculating effect size were excluded as well.

### 2.3. Study Selection and Data Extraction

Two reviewers independently screened the studies by title and abstracts and omitted nonrelevant studies. Then, both reviewers independently read the full text of each study, and according to the established eligibility criteria, they decided if it should be included or not. Conflicts were resolved by reaching consensus between reviewers and if disagreements persisted; a third author reviewed the study and made the final decision. Afterward, the reviewers independently extracted the relevant information such as first author, publication year, country of the study carried out in, type of the study, sample size, participants' age and gender distribution, and QOL questionnaire and scores from each study. Microsoft Excel was used to record the extracted information.

### 2.4. Quality Appraisal

The risk of bias in the included studies was assessed using the Joanna Briggs Institute (JBI) critical appraisal checklist for quasi-experimental and cross-sectional studies [23]. A cutoff of five positive items was used as the minimum quality for inclusion in the final analysis; this threshold provides a good balance between inclusivity and quality assurance. Studies that met this cutoff were deemed to have a low risk of bias and were therefore included in the final analysis. Risk of bias for each study was assessed by two independent reviewers. Any disagreements were resolved by discussion and in necessary cases by consulting a third reviewer. Also, reviewers took into account other factors that could affect the results of studies, such as sources of funding and conflict of interest, ensuring the results if this systematic review is reliable and robust.

### 2.5. Statistical Analysis

Intervention effects were determined using standardized mean differences (SMDs). The SMDs were calculated by subtracting pretreatment values from posttreatment values. Individual study weights were calculated as the inverse of the variance. Comparing pre- and postintervention QOL was done in three subgroups of follow-up time, i.e., 1, 3, and ≥ 6 months follow-ups. We also calculated the SMDs for comparing the QOL between patients who have undergone LVC versus emmetropes. Weighted averages and 95% confidence intervals were pooled using a random-effects model. The *I*^2^ statistic was calculated to determine the between-study statistical heterogeneity [24]. We also conducted a meta-regression analysis using the meta-reg command to assess the relationship between patients' age, refractive error, and follow-up time with SMDs. Publication bias was assessed with a funnel plot and Begg's test. Statistical analyses were performed using STATA version 17 (StataCorp LP, College Station, TX, USA) software. Two-tailed significant probability was considered less than 0.05.

## 3. Results

### 3.1. Search Results and Studies Characteristics

**Figure 1:**
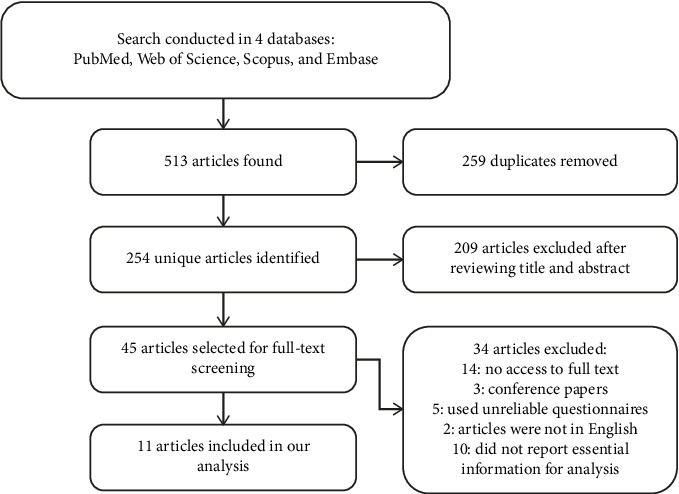
Flowchart of literature selection.

A total of 513 records were included in this review, and after removing duplicates, 254 articles remained. Following the screening of titles and abstracts, 209 of them were excluded due to irrelevant content. Finally, 45 articles were selected for full-text evaluation, and after a thorough assessment, 11 studies were selected for meta-analysis. The PRISMA flowchart for this review is shown in Figure 1.

**Figure 2:**
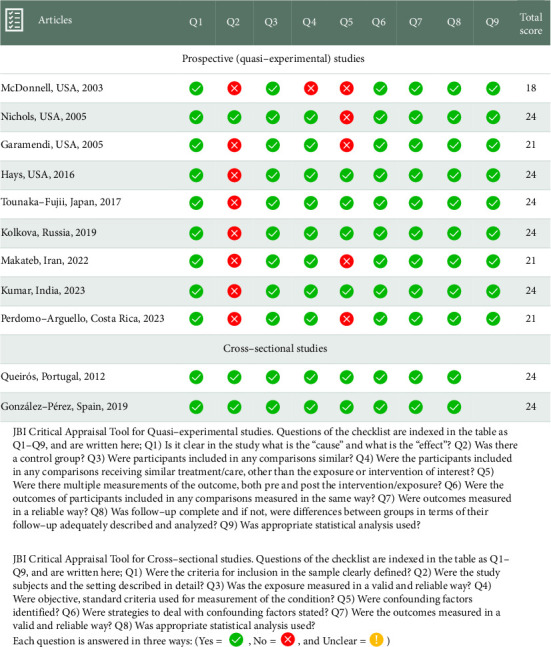
Quality appraisal assessment of included studies.

Table 1 summarizes the characteristics of the studies in the meta-analysis. Articles were published in various journals from 2003 to 2023 and conducted in Portugal, Iran, Spain, China, Japan, Russia, the United States of America, India, and Costa Rica. A total of 1753 participants, comprising of 1644 patients and 109 controls, were examined in these studies. The participants' mean age were 32.35 (95% CI: 28.09, 36.62) years. Females comprised 52.4% of all subjects. All of the studies used validated questionnaires such as NEI RQL-42 and 25, VFQ-25, QIRC, and SF-36 (mental component summary). In one study, FemtoLASIK and small-incision lenticule extraction (SMILE) were studied. Also, one study was conducted on patients who have undergone PRK. Intervention in the rest of the included studies was conventional LASIK.  Figure 2 provides a clear and concise overview of the quality of the included studies assessed by the JBI checklist for quasi-experimental and cross-sectional studies. Follow-up period ranged from 1 to 12 months. Some studies assessed the QOL at multiple intervals.

### 3.2. Effect of LVC on QOL in Pre–Post Design Studies

This group of articles was published from 2003 to 2023. A total of 1579 patients were included in these studies, of which 52.3% were females. QOL of patients was analyzed at three follow-up intervals: 1 month, 3 months, and 6 months.

**Figure 3:**
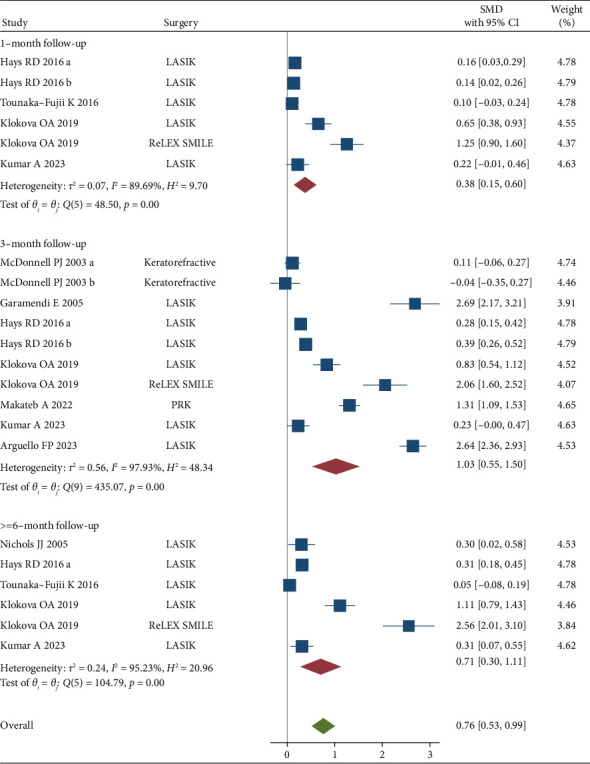
Forrest plot of standardized mean difference (SMD) and 95% CIs of mean QOL of before and after laser refractive surgeries. PROWL trial conducted by Hays et al. [27] had two groups; PROWL-1 was a single center study consisting of US navy personnel. PROWL-2 was a multi-center-study that recruited civilian participants: (a) PROWL-1 and (b) PROWL-2). Also, McDonnell et al. [20] reported their results separately for myopic and hyperopic patients who have undergone LVC surgery: (a) myopic and (b) hyperopic patients). These separate groups are represented by “a” and “b” in the figure (LASIK = laser-assisted in situ keratomileusis, PRK = photorefractive keratectomy, and SMILE = small incision lenticule extraction).

LVC improved QOL of patients at one (SMD = 0.38, 95% CI: 0.15, 0.60), three (SMD = 1.03, 95% CI: 0.55, 1.50), and six months or more after surgery (SMD = 0.71, 95% CI: 0.30, 1.11) (Figure 3).

**Figure 4:**
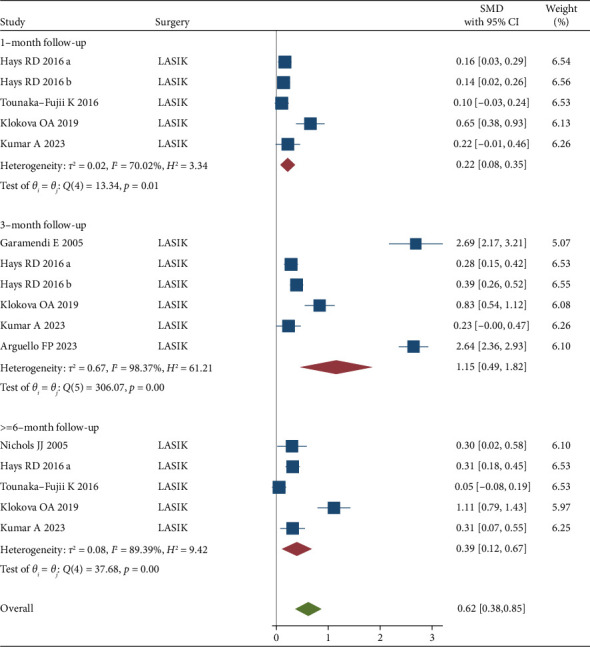
Forrest plot of standardized mean difference (SMD) and 95% CIs of mean QOL of before and after LASIK surgeries (LASIK = laser-assisted in situ keratomileusis).

There was evidence of heterogeneity among included studies (*I*^2^ > 70%). In a sensitivity analysis, excluding studies to LASIK surgeries, the results did not change substantially (Figure 4).

In the meta-regression analysis, patients' age (*β* = −0.06, 95% CI: −0.11, −0.01) showed an inverse significant association with the effect size, indicating that the QOL improvement was lower in older patients compared to younger ones. However, patient's baseline refractive error (*β* = 0.14, 95% CI: −0.01, 0.28), follow-up duration (*β* = 0.14, 95% CI: −0.42, 0.15), and study year (*β* = 0.04, 95% CI: −0.01, 0.09) did not show any significant association with the effect size (*p* > 0.05).

**Figure 5:**
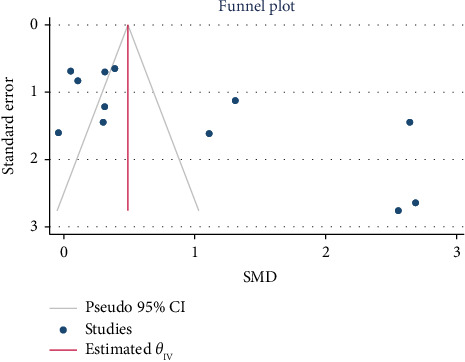
Funnel plot regarding SMD of the effect of surgery on quality of life at the longest follow-up time.

**Figure 6:**
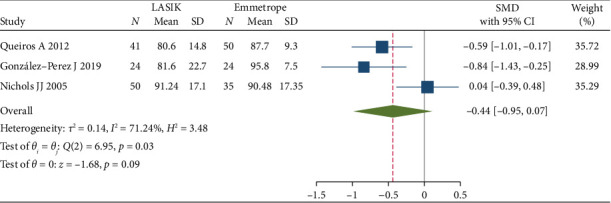
Forrest plot of standardized mean difference (SMD) and 95% CIs of mean QOL of the patients underwent LASIK surgeries vs. emmetropes (LASIK = laser-assisted in situ keratomileusis).

There was no evidence of publication bias between all included studies at the longest follow-up time (Begg's *p*=0.064) (Figure 5). Also, in a trim-and-fill analysis, no study was added or deleted (see Figure 6).

### 3.3. Comparing the QOL Between Patients Who Have Undergone LASIK Versus Emmetropes

This group of articles was published from 2005 to 2019. A total 109 subjects with age distribution of 29.2 ± 8.1 (mean ± SD) were enrolled in the control groups, where 52.2% of them were females. The intervention group consisted of 115 patients with age distribution of 34.67 ± 7.8 (mean ± SD), where 54% of them were females.

There was no significant difference between QOL of patients after LASIK and QOL of emmetropic patients (SMD = −0.44, 95% CI: −0.95, 0.07, *p* value: 0.09).

## 4. Discussion

Our comprehensive review of various studies revealed a promising trend: patients who have undergone LVC reported substantial improvements in their QOL. Our findings also suggest that QOL in patients after LVC was not inferior to emmetropes. These findings underscore the transformative potential of LVC in enhancing visual performance and overall life satisfaction, especially in younger patients where this improvement was more pronounced.

There are multiple criteria proposed for evaluating the efficacy and safety of refractive surgical procedures. Uncorrected and best CVA are the most commonly used metrics of efficacy and safety, respectively [33, 34]. In addition to that, the residual refractive error is used as a measure of efficacy and precision [35, 36]. However, these metrics fail to differentiate individuals who experience significant issues, despite a reasonable level of VA [20]. QOL questionnaires are one of the available tools used to assess the effectiveness of a patient's vision and their subjective experience. Evaluation of the patient's QOL alongside objective measurements such as VA, through the questionnaires, provides further insights into patient satisfaction and expectations [11]. This study took into account the surgical methodology, the type of questionnaire used, and the outcomes reported by the patients in response to the questionnaire. The results indicated that the average QOL score improved after any laser refractive surgery compared to before [1, 11, 20, 25–32]. This improvement in QOL was consistent across different types of questionnaires used and seemed be present globally.

Refractive surgery is commonly associated with the experience of anxiety, irritation, sensitivity to light, pain, diminished low-light vision, haloes, and glare; these symptoms are primarily attributable to the dramatic changes in the corneal shape, ocular aberrations, or dry eye, which is particularly noticeable in the first month after surgery [1, 26, 28]. Prevalence of dry eye disease in general population is about 8% [37]. According to previous studies, 36%–75% of patients experience various degrees of dry eye symptoms in the immediate period after LASIK; however at 3 months, only 4% of patients had severe symptoms [38]. In comparison, dry eye symptoms are observed in 50% of patients wearing contact lenses [39]. Our findings also suggest that, with diminishing these transient symptoms after the first month, QOL of patients continues to improve. The improvement in psychosocial feelings may be partly attributed to the elimination of inconveniences, related to the use of spectacles or contact lenses; inconveniences such as glasses getting dirty or scratched, and contact lenses falling out or getting lost. The removal of these limitations can markedly improve various aspects of life, including social roles, job performance, intimate relationships, and the ability to communicate effectively with coworkers and friends. This can lead to increased self-confidence and improved self-perception, which in turn can enhance the QOL scores [25, 40–42].

In pooling the results of three studies with emmetropic controls [1, 25, 29], the mean QOL after surgery was not substantially different compared to emmetropes. However, in one study investigated by Javier Gonzalez, LASIK group outcomes were lower than emmetropes, most probably because of haloes and glare after surgery in this study [29]. This is highlighted by the lower scores that they reported in areas such as diurnal fluctuations, glare, expectations, and concerns about visual issues, compared to the emmetropic group. This could be attributed to high spherical aberration caused by the corneal reshaping process. However, the overall results indicated no significant difference in the average QOL between patients after surgery and emmetropes. This is a very impactful conclusion because it revealed that refractive surgery could improve patients' QOL to a level comparable to that of an emmetrope.

Patients after refractive surgery typically have little or no trouble using nonprescription sunglasses, seeing when walking, swimming, or exercising, and they do not have to worry about spectacles or contact lenses before traveling. Alleviation of these issues increases the QOL of patients after surgery. However, other factors should be considered as well, such as cognitive dissonance. Patients who have undergone surgery could justify this choice by reporting that the outcome was successful. Although this probably plays a role, its impact is likely to be negligible when using a questionnaire, where the way to distort the measurement of the outcome may be less pronounced [29, 43].

One major limitation of our review was lack of access to preoperative data of some related studies that led them to not being included in the study, which resulted in a smaller study population. Other noteworthy limitation was not including publications in languages other than English. Also, there was significant heterogeneity observed in included studies.

## 5. Conclusion

We surmised that patients undergoing LVC experience significant improvements in QOL and achieve comparable QOL to individuals with emmetropia. Also, this improvement is more prominent in younger patients.

## Figures and Tables

**Table 1:** Basic characteristics of included studies.

First author	Year	Country	Type	Type of LVC	Questionnaire of QOL	Intervention group	Control group	Follow-up (months)
McDonnell et al. [20]	2003	California, USA	Prospective	LASIK	NEI-RQL_42	185	—	3
Nichols et al. [25]	2005	Ohio, USA	Prospective	LASIK	NEI-RQL_42	50	72	6
Garamendi et al. [26]	2005	Texas, USA	Prospective	LASIK	NEI-RQL_42	66	—	3
Queirós et al. [1]	2012	Portugal	Cross-sectional	LASIK	NEI RQL-42	41	50	3
Hays et al. [27]	2016	California, USA	Prospective	LASIK	NEI-RQL_42	511	—	1, 3, 6
Yuki et al. [28]	2017	Japan	Prospective	LASIK	HRQoL MCS	213	—	1, 6
González-Pérez et al. [29]	2019	Spain	Cross-sectional	LASIK, CRS	NEI RQL-42	24	24	12
Klokova et al. [11]	2019	Russia	Prospective	Femto LASIK, SMILE	QIRC	118	—	1, 3, 6
Makateb et al. [30]	2022	Iran	Prospective	PRK	QIRC	147	—	3
Kumar et al. [31]	2023	India	Prospective	LASIK	NEI-RQL_42	71	—	1, 3,6
Perdomo-Arguello et al. [32]	2023	Costa Rica	Prospective	LASIK	NEI VFQ-25	218	—	3

Abbreviations: CRS = corneal refractive surgery, HRQoL MCS = health-related quality of life mental component summary, LASIK = laser -assisted in situ keratomileusis, LVC = laser vision correction, NEI-RQL_42 = National Eye Institute Refractive Error Quality of Life Instrument-42, NEI VFQ-25 = National Eye Institute Visual Function Questionnaire 25, PRK = photorefractive keratectomy, QIRC = quality of life impact of refractive correction, and QOL = quality of life.

## Data Availability

The data supporting the findings of this study are accessible upon request from the corresponding authors.
